# External validation of the GRACE risk score in patients with myocardial infarction in Hungary

**DOI:** 10.1016/j.ijcha.2023.101210

**Published:** 2023-04-24

**Authors:** Tamás Ferenci, Péter Hári, Péter Vájer, András Jánosi

**Affiliations:** a Physiological Controls Research Center, Óbuda University, Budapest, Hungary; b Department of Statistics, Corvinus University of Budapest, Budapest, Hungary; c Delta Group, Budapest, Hungary; d Gottsegen National Cardiovascular Center, Budapest Hungary, Hungarian Myocardial Infarction Registry, Budapest, Hungary

**Keywords:** GRACE risk score, Validation, Acute myocardial infarction, Prognosis, Outcome

## Abstract

**Background:**

Literature confirms that the Global Registry of Acute Coronary Events (GRACE) risk score provides a better risk evaluation than clinical judgment in patients with acute myocardial infarction. We aimed to externally validate the GRACE risk score in unselected patients with myocardial infarction in Hungary.

**Methods:**

Data from the comprehensive Hungarian Myocardial Infarction Registry (HUMIR), a national registry that collects data on consecutive acute myocardial infarction (AMI) patients, were used. Hospitals registered 102,939 infarction events in the HUMIR between January 1, 2014, and December 31, 2020. The data required to calculate GRACE risk score were available for 75,199 events. We studied the 6-months, 1-year, and 3-year outcomes. We calculated widely used metrics to characterise calibration (calibration curve, calibration intercept and slope, Eavg, Emax, and E90) and discrimination (*c*-score, equivalent to AUC, and Somer's *D_xy_*).

**Results:**

The risk of low-risk patients was underestimated, and the risk of high-risk patients was overestimated. However, the deviation was small, especially for the three-year survival (E90 was 0.15, 0.22, and 0.08). Discrimination was good, with an AUC of approximately 0.8, and was very similar in all the periods.

**Conclusions:**

These data confirmed the usefulness of GRACE risk score in selecting high-risk patients with myocardial infarction in the Hungarian population.

## 1 Introduction

Acute coronary syndrome (ACS) is one of the leading causes of death worldwide. Invasive management has the potential to improve the prognosis of patients with ACS. Current guidelines [1], [2] emphasise the importance of early catheter revascularisation, i.e., percutaneous coronary intervention (PCI) in patients with ST-elevation myocardial infarction (STEMI). In the case of non-ST-elevation myocardial infarction (NSTEMI), PCI is indicated in high/moderate-risk patients. For patients with NSTEMI infarction, adequate risk assessment and subsequent clinical management are thus mandatory to optimise patient outcomes. However, assessing the risk of patients with NSTEMI with the diverse clinical picture is challenging and imprecise [3], [4]. Literature data confirms that the Global Registry of Acute Coronary Events (GRACE) risk score gives better risk evaluation than clinical judgment [3], [4]. However, in everyday practice, guideline-directed care is inversely related to the estimated risk of patients with NSTEMI, which is called the 'risk-treatment paradox' [1]. There are validation studies [1], [5] of this score, but publication using the East European population sample is missing. Our present work investigates the validity of GRACE risk score in Hungary using data from a mandatory myocardial infarction registry covering seven years with 102,939 events.

## 2 Methods

The Hungarian Myocardial Infarction Registry (HUMIR) is a national online registry that collects data on consecutive unselected acute myocardial infarction (AMI) patients. Data collection is mandatory in Hungary, according to the statute of CCXLVI. /2013 of Hungary. Data capture covered 178 structured variables, including prehospital data, medical history, hospital medications, and coronary interventions. The registry database included 91.3% of all AMI cases in 2020 compared with the national healthcare provider reimbursement dataset. The data were continuously checked and validated. In addition, follow-up outcome data, including vital status and repeated hospitalisations, were regularly obtained from the electronic database of the national healthcare insurance provider. Hospitals registered 102,939 infarction events in the HUMIR between January 1, 2014, and December 31, 2020. Age, prehospital resuscitation, pulse rate, systolic blood pressure, ST changes, Killip class at admission, initial cardiac biomarker, and serum creatinine were used to calculate the score. The prediction of in-hospital mortality was calculated according to Granger [3], and the six months, one-year, and three-year scores were calculated using the Fox model [4]. Follow-up data on death were available for all patients as of June 30, 2021. The protocol of the study is in accordance with the Declaration of Helsinki, and it was approved by the Hungarian National Committee of Health Research Ethics.

## 3 Statistical Methods

Categorical variables are presented as percentages (counts), and continuous variables are presented as lower quartile/median/upper quartile and mean ± standard deviation. Survival was calculated using the non-parametric Kaplan–Meier estimator [6].

Calibration and discrimination are essential for validating the prognostic score [7], [8], [9], [10], [11]. Here, we calculate the calibration curve, which shows the actual vs. predicted outcome (without binning, using spline smoothing), and present the average (Eavg) and maximum (Emax) of the difference from the ideal curve along with the 90th percentile of the differences (E90) to describe it numerically. We also calculated the calibration intercept (to see systematic under- or over-prediction, i.e., calibration-in-the-large) and calibration slope.

For discrimination, we calculated the *c*-score, equivalent to the area under the receiver operating characteristic curve (AUC), which is identical to Somer's *D_xy_* rank correlation between the predicted probability and the actual outcome.

Calculations were performed using the R statistical software version 4.1.2 (R Core Team, Vienna, Austria) using the rms package version 6.2–0.

## 4 Results

Between 01.01.2014 and 31.12.2020, 102,939 AMI events were registered in the HUMIR, with the data required to calculate GRACE risk score being available for 75,199 events (73.1%). Events where GRACE score could not be calculated due to missing data were excluded from the analysis (26.9%). The 6-month, 1-year, and 3-year outcomes were calculated. Table 1 summarizes the medical history, comorbidities, presentation characteristics, and variables necessary for the GRACE risk score calculation. Of note, 81% had hypertension, 35% had diabetes mellitus, 24% had a previous myocardial infarction. Most patients (95%) were in Killip classes I and II. The initial renal function was abnormal in 21% of the patients (defined as a serum creatinine level above 110 umol/l, i.e., 1.24 mg/dL), and the cardiac biomarkers were abnormal in almost all cases (99%). Percutaneous coronary intervention was performed in 71% of the patients.

**Table 1:** Characteristics, factors used for GRACE calculation, diagnostic and therapeutic interventions of events. Continuous variables are presented as “median (lower quartile – upper quartile), mean ± standard deviation”, categorical variables are presented as “count (percentage)”.

	Number of events with non-missing value	
Age	75,199	67 (58 – 76), 66.4 ± 12.8
Female	75,199	29,520 (39.2%)
STEMI	75,199	34,964 (46.5%)
Medical history		
Hypertension	74,122	59,779 (80.6%)
Stroke	73,018	6,707 (9.2%)
Myocardial infarction	73,368	17,235 (23.5%)
CABG	73,616	3,810 (5.2%)
PCI	73,607	15,468 (21.0%)
Comorbidities		
Diabetes mellitus	73,440	25,803 (35.1%)
Peripheral arterial disease	70,682	10,251 (14.5%)
Active smoker	44,699	20,010 (44.8%)
Parameters on admission		
Prehospital reanimation	74,089	2,767 (3.7%)
Killip 1	75,199	63,134 (84.0%)
Killip 2	75,199	8,342 (11.1%)
Killip 3	75,199	2,457 (3.3%)
Killip 4	75,199	1,266 (1.7%)
Systolic blood pressure	75,199	134 (120 – 150), 134.9 ± 25.8
Pulse rate	75,199	800 (70.0 – 93), 83.0 ± 19.8
Serum creatinine	75,199	89.9 (80.9 – 102.9), 102.9 ± 61.2
Creatinine > 110 umol/l	75,199	15,597 (20.7%)
Positive biomarker	75,199	74,120 (98.6%)
ST-change	75,199	59,434 (79.0%)
Cardiogenic shock	75,199	1,542 (2.1%)
Diagnostic and therapeutic interventions		
Coronarography	75,199	64,673 (86.0%)
PCI	75,199	53,623 (71.3%)

**Fig. 1:**
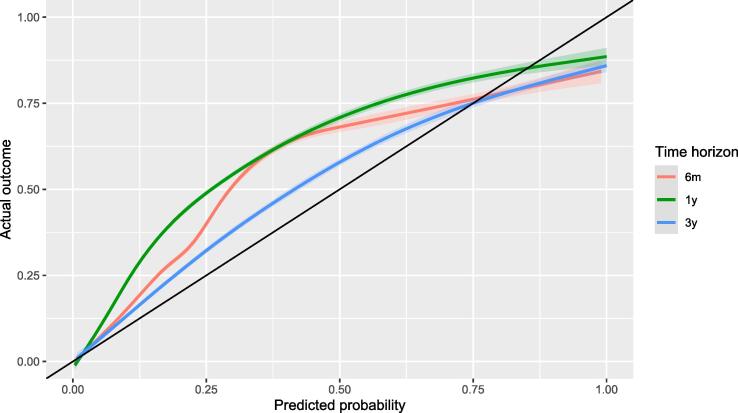
Calibration curves (predicted probability of survival vs. actual survival) estimated with spline-smoothing for all three time horizons.

Fig. 1 shows the calibration curves at different time horizons, i.e., it gives the actual risk of the patients for a given predicted risk (that is, low risk patients are on the lower left part, high risk patients are on the upper right part with the diagonal black line indicating perfect prediction when the actual risk equals the predicted risk). Table 2 lists the numerical values of the validation metrics which give a quantitative characterization of this calibration curve. Calibrations showed a similar pattern in all the periods: the risk of low-risk patients was underestimated, and the risk of high-risk patients was overestimated. Interestingly, this was more apparent in the shorter time span (the maximum difference was 0.20 at six months and 0.23 at one year, but only 0.11 at three years; the same pattern is true for Eavg and E90). Nevertheless, the calibration slope is worse at three years, but the calibration intercept is the opposite; it is better for three years than for the shorter periods.

**Table 2:** Numerical values of different indicators of discrimination (D_xy_, AUC, or *c*-index, and *R^2^*) and calibration (calibration intercept and slope, and Emax, E90, and Eavg distance metrics) for all the three periods.

	**6 months**	**1 year**	**3 years**
D_xy_	0,61225	0,637292	0,589794
*c* (AUC)	0,806125	0,818646	0,794897
*R^2^*	0,241233	0,205493	0,238306
Brier	0,122364	0,14105	0,16177
Intercept	0,718455	1,021026	0,005905
Slope	1,099231	1,067366	0,751439
Emax	0,203239	0,234247	0,106194
E90	0,157034	0,217474	0,077721
Eavg	0,056187	0,091109	0,046625

As far as discrimination is concerned, performance is similar in all three periods. In terms of AUC, that is, *c*-index, and therefore Somer's *D_xy_*, the results are almost identical (0.81 at six months, 0.82 at one year, and 0.79 at three years). All values indicated reasonably good discrimination.

Overall, the predictive performance was good and very similar in all the periods.

## 5 Discussion

Our study examined the effectiveness of GRACE risk score in a large number of patients. To our best knowledge, this is the first validation of the GRACE score in Central Europe using a large, unselected, registry-based population.

The predictive performance of the risk score was good for all the periods. Calibration was slightly better at three years, with a worse performance observed at the shorter periods, but discrimination was almost identical and good, with an AUC of approximately 0.8 for all the three periods.

Similar to the present study, the applicability of GRACE risk score in different populations has been confirmed in several other investigations [12], [13], [14]. The significance of our research is highlighted by the fact that validation has not yet taken place in a population sample of Central and Eastern Europe. Several studies have compared different prognostic scores. Parco et al. [15] compared the GRACE risk score, Acute Coronary Treatment and Interventions Outcomes Network (ACTION), and National Cardiovascular Data (NCDR) risk models and found that ACTION and NCDR were more effective than GRACE risk score in predicting in-hospital mortality. In the case of ST-elevation myocardial infarction TIMI (thrombolysis in myocardial infarction), CADILLAC (controlled abciximab and device investigation to lower late angioplasty complications), and GRACE risk score, all three prognostic scores were excellent in predicting 30-day and one-year mortality [16]. Rahmani et al. [17] compared the GRACE risk score and Synergy between PCI with Taxus and Cardiac Surgery (SYNTAX) scores in patients with the acute coronary syndrome. GRACE risk score showed a moderate but significant association with SYNTAX score. The “risk-management paradox” is a common experience: PCI is more frequent than necessary in lower-risk patients and less common in high-risk patients [18]. Dondo et al. [19] investigated the extent and consequences of nonadherence to guideline-indicated care across a national health system (Myocardial Ischemia National Audit Project) in patients with non-ST-elevation myocardial infarction. Coronary angiography was missed in 46.3% of patients and had the most substantial impact on reduced survival. Everett et al. [20] recently started a parallel-group cluster randomized control trial that allocated hospitals in a 1:1 ratio to manage non-ST-elevation myocardial infarction. One group evaluated the patient's risk according to clinical evaluation only, in contrast, the other group used clinical evaluation and GS risk score for risk assessment and an indication of invasive strategy.

The main strength of our study is the very high (>70,000) sample size, which is a magnitude higher than most validation studies in the literature and its completely unselected nature representing the real-life experience in Hungary over an eight-year-long period. Of note, reliable follow-up data was available for every patient. Such validations are rare generally, and to our best knowledge, none is available from the East European region. The length of the follow-up in the present study is itself unique: very few studies addressed the issue of validating the GRACE risk score beyond a 1 year follow-up, even those that did, typically had a lower sample size (Kozieradska et al investigated 5 year mortality, but only with 505 patients [21], Tang et al investigated 4 year mortality with 1,143 patients [22]). To the best of our knowledge, this is the first investigation in which the GRACE score was analyzed in terms of 3-year survival using a very large number of myocardial infarction patients.

The main limitation of our study concerns the missing values: in 26.9% of the registered events, at least one data was not reported that would have been needed to calculate the risk score. We cannot be sure if this represents missingness completely at random, or if the missingness was related to the actual prognosis of the patient, in which case it biases our results (selection bias). Data were not extracted from primary sources (e.g., laboratory results were not extracted from the hospital information system), but rather manually entered by the attending physician. This leaves room for clerical errors and negligence, but this is unlikely to be systematical causing bias.

## 6 Conclusions

Our data confirm that GRACE risk score is an appropriate tool: the calibration, while not perfect, was acceptable, and discrimination demonstrated good capability to select high-risk patients with non-ST-elevation myocardial infarction in the Hungarian population. Furthermore, our results support the current guidelines where the use of GRACE risk score has a class IIa and level B recommendations.

## 7 Funding statement

This research did not receive any specific grant from funding agencies in the public, commercial, or not-for-profit sectors.

## Declaration of Competing Interest

The authors declare that they have no known competing financial interests or personal relationships that could have appeared to influence the work reported in this paper.
